# Measuring Indigenous homelessness: Findings from Our Health Counts Toronto

**DOI:** 10.17269/s41997-024-00974-7

**Published:** 2024-11-12

**Authors:** Stephanie McConkey, Julia Iannace, Marcie Snyder, Cheryllee Bourgeois, Janet Smylie

**Affiliations:** 1 Department of Epidemiology, Dalla Lana School of Public Health, University of Toronto, Toronto, ON Canada; 2 Well Living House, St. Michael’s Hospital, Unity Health Toronto, Toronto, ON Canada; 3 Seventh Generation Midwives Toronto, Toronto, ON Canada; 4 Dalla Lana School of Public Health and Department of Family and Community Medicine, Faculty of Medicine, University of Toronto, Toronto, ON Canada

**Keywords:** Indigenous peoples, North American, Community-based participatory research, Population health, Housing, Housing instability, Peuples autochtones, Nord-Américains, Recherche participative basée sur la communauté, Santé de la population, Logement, Instabilité en matière de logement

## Abstract

**Objectives:**

Our Health Counts (OHC) Toronto, an Indigenous population database which addresses gaps in urban health information, was used to measure Thistle’s (2017) 12 dimensions of Indigenous homelessness. Using this framework, we examine the sociodemographic characteristics of First Nations, Inuit, and Metis (FNIM) adults living in Toronto, the 12 dimensions as experienced by this population, and the distinctions between FNIM adults who were and those who were not experiencing physical homelessness.

**Methods:**

Respondent-driven sampling (RDS)-II proportions and 95% confidence intervals were produced from the database (*n* = 915 FNIM adults) to describe key sociodemographic characteristics of the population and to estimate the proportion and number of dimensions of Indigenous homelessness experienced by FNIM adults. Results were compared between those who were and those who were not living physically homeless.

**Results:**

This study shows that 27.3% of FNIM adults in Toronto were living physically homeless. The proportion of homelessness was significantly higher among males, adults aged 26 to 54, and unemployed individuals. Using the OHC database, 7 of the 12 dimensions were measurable. Almost all FNIM adults had experienced one or more of the 7 measurable dimensions. The most common were cultural disintegration and loss, mental disruption and balance, contemporary geographic separation, and relocation and mobility. These dimensions were significantly more common among FNIM adults experiencing physical homelessness.

**Conclusion:**

Results show that FNIM adults living physically homeless are more likely to experience other dimensions of homelessness. Using existing data, 5 of the 12 dimensions were not measurable. This points to a critical need to develop new survey tools to fully understand the historical, environmental, social, political, spiritual, and emotional factors that influence pathways into homelessness among FNIM populations.

## Résumé

**Objectifs:**

Notre santé compte (NSC) Toronto, une base de données sur la population autochtone qui comble des lacunes dans les informations sur la santé en zone urbaine, a servi à mesurer les 12 dimensions de l’itinérance autochtone de Thistle (2017). À l’aide de ce cadre, nous avons étudié les caractéristiques sociodémographiques d’adultes des Premières Nations, inuits et métis (PNIM) vivant à Toronto, les 12 dimensions telles qu’elles s’appliquent à cette population et les distinctions entre les adultes PNIM étant ou non en situation d’itinérance physique.

**Méthode:**

À partir de la base de données de NSC (n = 915 adultes PNIM), nous avons produit des pondérations EFR-II (échantillonnage en fonction des répondants) et des intervalles de confiance de 95% pour décrire les principales caractéristiques sociodémographiques de la population et estimer la proportion et le nombre de dimensions de l’itinérance autochtone s’appliquant aux adultes PNIM. Nous avons comparé les résultats obtenus pour les personnes étant et n’étant pas en situation d’itinérance physique.

**Résultats:**

L’étude montre que 27,3% des adultes PNIM de Toronto étaient en situation d’itinérance physique. Le taux d’itinérance était sensiblement plus élevé chez les hommes, les adultes de 26 à 54 ans et les personnes sans emploi. Sept des 12 dimensions de l’itinérance autochtone étaient mesurables à l’aide de la base de données de NSC. Une ou plusieurs de ces 7 dimensions mesurables s’appliquaient à presque tous les adultes PNIM. Les plus courantes étaient la désintégration culturelle et la perte culturelle, la perturbation mentale et le déséquilibre mental, la séparation géographique contemporaine, et la relocalisation et la mobilité. Ces dimensions étaient beaucoup plus courantes chez les adultes PNIM en situation d’itinérance physique.

**Conclusion:**

Nos résultats montrent que les adultes PNIM en situation d’itinérance physique sont plus susceptibles d’être aux prises avec d’autres dimensions de l’itinérance. Cinq des 12 dimensions n’étaient pas mesurables à l’aide des données existantes. Il est donc absolument nécessaire d’élaborer de nouveaux outils de sondage pour bien comprendre les facteurs historiques, environnementaux, sociaux, politiques, spirituels et affectifs qui influent sur les parcours d’itinérance dans les populations PNIM.

## Introduction

Indigenous people living in urban and related homelands experience notably high rates of homelessness, precarious housing, overcrowding, and unsafe housing (Brar et al., 2020; Firestone et al., 2018). More specifically, rates of homelessness are eight times higher for Indigenous populations living in cities than for the general population (Belanger, 2013). In 2015–2016, approximately 1 in 3 Indigenous adults in Toronto were living physically homeless or precariously housed (Firestone et al., 2018). Using this information and current population estimates (Rotondi et al., 2017; Smylie et al., 2022), approximately 24,000 Indigenous adults were physically homeless or precariously housed in Toronto.

As a result, disparities in access to safe and affordable housing exist for Indigenous people living in cities. Research shows that Indigenous people are commonly subjected to racism and discrimination in the housing market (Allan & Smylie, 2015; Corrado Research and Evaluation Associates Inc., 2003), thereby impacting their ability to secure housing for rental or ownership. Housing quality is also a concern, with common reports of defective plumbing, poor electrical wiring, and structural issues among housed Indigenous people (Firestone et al., 2018; Rodrigues et al., 2020). Barriers to safe, stable housing can lead to overcrowded living conditions and increased risk of physical homelessness (Belanger et al., 2012). Experiences of homelessness are often compounded by systemic and socio-economic factors, as well as mental, emotional, physical, spiritual, and behavioural factors (Gaetz et al., 2012). A myriad of challenges ultimately impact pathways into homelessness among Indigenous populations in urban and related homelands and contribute to intergenerational income disparities and involuntary migration (Belanger, 2013; Distasio et al., 2013). These challenges are rooted in ongoing systemic racism and include substance use, cultural detachment, child protection agency involvement, experiences of racism, violence, sexual abuse, decreased autonomy, lack of social supports, family stress, employment status, and landlord racism (Belanger, 2013; Christensen, 2013; Distasio et al., 2013; Gabriel et al., 2022). Cycles of intergenerational homelessness due to social factors and collective community trauma perpetuate conditions related to precarious housing and homelessness (Belanger, 2013; Menzies, 2006).

Canadian definitions of homelessness focus on an individual’s living situation, specifically whether a person is unsheltered, living in emergency housing, provisionally accommodated, or at risk of experiencing housing instability (Belanger, 2013; Gaetz et al., 2012). Western perspectives of homelessness fail to acknowledge the spiritual, cultural, and emotional aspects that contribute to a sense of home or homelessness (Christensen, 2013; Thistle, 2017) and lack consideration for the intersecting historical, environmental, social, and political factors that drive physical homelessness among Indigenous populations (Belanger et al., 2012; Belanger, 2013; Menzies, 2006, 2008). To define and measure Indigenous homelessness, the concept must be qualitatively distinguished from western definitions of homelessness.

To address these gaps, Thistle (2017) defines Indigenous homelessness using the 12 dimensions of Indigenous homelessness, which encompass Indigenous worldviews and understandings of home that extend beyond a physical habitation structure. Only one dimension is dedicated to physical homelessness, while the remaining 11 focus on the historical and contemporary displacement of Indigenous peoples due to the longstanding legacy of colonialism that has harmed connections to self, identity, family, community, culture, place, land, water, animals, and language (Thistle, 2017; Thistle & Smylie, 2020). This framework highlights a significant need to revise how academics and policymakers define and measure homelessness to accurately reflect Indigenous epistemologies and the unique experiences of Indigenous people (Belanger et al., 2012; Christensen, 2013; Thistle, 2017).

Drawing on existing population data sources of Indigenous people living in Toronto, this paper explores the 12 dimensions of Indigenous homelessness (Thistle, 2017) to further understand the historical, environmental, social, political, spiritual, and emotional factors that influence pathways into physical homelessness among Indigenous people living in urban and related homelands. The objectives of this paper are therefore to use this data source and the 12 dimensions of Indigenous homelessness as a framework to (1) understand key sociodemographic characteristics of First Nations, Inuit and Metis (FNIM) adults experiencing physical homelessness; (2) produce population-based estimates and counts of the total number of dimensions experienced by FNIM adults in Toronto; and (3) compare the difference in proportions of key sociodemographic factors and dimensions of homelessness between FNIM adults who were and those who were not experiencing physical homelessness at the time of data collection.

## Methods

### Indigenous research methodologies

Under the guidance of a local reference group comprised of Indigenous and allied health and social service providers in Toronto, Seventh Generation Midwives Toronto and the Well Living House completed an Our Health Counts (OHC) study to collect health assessment data on the FNIM population living in Toronto. The OHC study was rooted in Indigenous research practices and protocols and is described in more detail elsewhere (Firestone et al., 2022; Smylie et al., 2024). The St. Michael’s Hospital Research Ethics Board granted ethics approval for this research project.

### Study population

Secondary data analysis was completed using the OHC Toronto dataset, which includes *n* = 915 self-identified FNIM adults (≥ 15 years of age) who lived, worked, or accessed health or social services in Toronto between March 2015 and March 2016. Respondent-driven sampling (RDS) was used to recruit FNIM participants and is further detailed in other OHC publications (Firestone et al., 2022; Rotondi et al., 2017).

### Measures

Authors mapped out the OHC adult survey tool in its entirety to match relevant OHC topics and questions related to the 12 dimensions of Indigenous homelessness (Table 1). This mapping exercise showed that some dimensions did not have relevant measures available in the data source and some had multiple relevant questions. All dimensions are defined below; however, only measurable dimensions were included in the statistical analysis.

**Table 1:** Mapping the 12 dimensions of Indigenous homelessness (Thistle, 2017) to relevant OHC Toronto indicators

#	Dimension	Definition	Question topics	Relevant OHC questions
1	Historic displacement	Displaced from pre-colonial Indigenous lands	Forced relocation to reserved lands or settlements	N/A
			Loss of Indigenous knowledge	N/A
2	Contemporary geographic separation	Displaced from Indigenous lands, post-colonialization	Residential schools	1. Were you ever a student at a federal residential school, or federal industrial school?
				2. Were any of the following members of your family ever a student at a federal residential school or federal industrial school?
			Sixties Scoop	3. Were you or other members of your family adopted between 1951 and 1970, during the Sixties Scoop?
			Racism/discrimination	4. Have you ever been treated poorly or unfairly because you are Indigenous?
			Disconnection to land	N/A
3	Spiritual disconnection	Separation from Indigenous worldviews and ways of being/doing	Disconnection to Indigenous spirituality:	
			Ceremonies	1. Do you participate in traditional Indigenous ceremony?
			Indigenous medicines	2. Do you use traditional Indigenous medicines or practices to maintain your well-being?
			Language	3. Do you speak an Indigenous language or languages?
			Traditional foods	4. In the past 12 months, how often have you eaten traditionally hunted/gathered/grown and/or country foods?
4	Mental disruption and imbalance	Imbalance of mental wellness as a result of history of colonization and marginalization	Mental health:	
			Mental health diagnosis	1. Have you ever been told by a health care worker that you have a psychological and/or mental health disorder?
			K-10 scale (positive screen)	2. K-10 scale
			PTSD (positive screen)	3. PC-PTSD scale
5	Cultural disintegration and loss	Disconnection to Indigenous culture and relationships	Disconnection to Indigenous culture and lack of/limited access to:	
			Ceremonies	1. Have you experienced any challenges in trying to access traditional Indigenous ceremonies?
			Traditional foods	2. Would you prefer eating more traditional/country foods than you can get?
			Land	3. How often do you feel strong in your relationship to land/Mother Earth?
			Kin and relationships (social network)	4. About how many close friends and close relatives do you have, that is, people you feel at ease with and can talk to about what is on your mind?
			Indigenous medicines	N/A
			Language	N/A
6	Overcrowding	The number of people per household exceeding national average	# of rooms in household	How many rooms are there in your home/place for staying? (not counting bathrooms, hallways, or office rooms)
			# of people living in household	Including yourself, how many people currently live/stay in your household?
				Create a derived variable: Overcrowding = > 1 person/room
			Multigenerational living	N/A
7	Relocation and mobility	Moving between community and other geographic places for access to work, health, and housing, among others	Geographic relocation	How many times have you moved in the past year?
			Reasons for relocating	N/A
8	Going home	Being unable to secure stable housing in home community as a result of being away from home community for a length of time (i.e., for school, employment, other)	Access to physical space in community (individual or family)	N/A
			Lateral violence	N/A
9	Nowhere to go	Lack of access to physical space/shelter	Physical homelessness	Which of the following best describes the type of residence you currently live in?
				Create a derived variable: physical homelessness = living on the streets OR living in transition
10	Escaping or evading harm	Leaving community due to feeling unsafe	Feeling unsafe in home community	N/A
			Lateral violence	N/A
11	Emergency crisis	Forced relocation due to natural disasters or other environmental factors	Experiences of natural disasters in home community	N/A
12	Climate refugee	Forced relocation due to impacts of climate change on the land (being unable to hunt, gather, or access water)	Impacts of climate change in home community	N/A

#### Nowhere to go (physical homelessness)

“Nowhere to go” homelessness, also referred to as physical homelessness throughout this article, is the only dimension that overlaps with western conceptualizations of homelessness (i.e., being unable to secure or access stable shelter) (Thistle, 2017). A derived variable was used to describe nowhere to go homelessness using measures about the type of residence that participants were residing in at the time of data collection. Participants who responded, “living on the streets” or “living in transition” to the question “which of the following best describes the type of residence you currently live in?” were categorized as experiencing nowhere to go homelessness/physical homelessness.

Key sociodemographic characteristics of the entire population were explored and stratified by the population living physically homeless and the population not living physically homeless (Table 2), as were the 12 dimensions of Indigenous homelessness (Table 3).

**Table 2:** Sociodemographic characteristics of FNIM adults in Toronto living and not living physically homeless

Characteristics	FNIM adults in Toronto (*n* = 915)		FNIM adults living physically homeless (*n* = 154)^a^		FNIM adults living physically housed (*n* = 734)^a^		Difference in proportions using RDS MOVER % (95% CI)
	*n*	RDS-II % (95% confidence interval)	*n*	RDS-II % (95% confidence interval)	*n*	RDS-II % (95% confidence interval)	
Indigenous identity							
First Nations	804	85.5 (81.5, 89.5)	133	94.9 (91.2, 98.6)	646	81.6 (76.7, 86.4)	13.3 (7.2, 19.4)*
Métis	89	13.4 (9.6, 17.3)	15	3.3 (1.1, 5.6)	72	17.5 (12.9, 22.3)	− 14.2 (− 19.5, − 8.9)*
Inuit	11	0.4 (0.0, 0.9)	-	0.3 (0.0, 2.1)	9	0.4 (0.0, 0.9)	− 0.1 (− 1.1, 1.3)
Multiple Indigenous identities	-	0.5 (0.0, 1.3)	-	0.5 (0.0, 1.1)	-	0.4 (0.0, 1.5)	0.1 (− 1.1, 1.4)
Other	-	0.2 (0.0, 0.5)	-	0.0 (0.0, 0.0)	-	0.0 (0.0, 0.0)	0.0 (0.0, 0.0)
Age							
Under 25	126	23.3 (18.6, 28.0)	16	17.1 (7.0, 27.2)	103	25.6 (20.3, 30.9)	− 8.5 (− 19.9, 2.9)
26 to 54	603	63.0 (57.4, 68.5)	118	79.7 (69.3, 90.2)	469	56.3 (50.1, 62.6)	23.4 (11.2, 35.6)*
55 +	186	13.8 (9.5, 18.1)	20	3.1 (0.0, 6.7)	162	18.0 (12.8, 23.3)	− 14.9 (− 21.0, − 8.5)
Gender							
Male	421	49.7 (44.0, 55.3)	105	65.7 (52.6, 78.7)	302	43.5 (37.2, 49.8)	22.2 (7.7, 36.6)*
Female	477	48.9 (43.1, 54.5)	45	32.3 (19.5, 45.1)	419	55.2 (48.9, 61.4)	− 22.9 (− 37.1, − 8.7)
Other	-	0.4 (0.0, 1.5)			5	0.7 (0.0, 1.9)	0.0 (0.0, 0.0)
Trans	-	1.0 (0.3, 1.7)	-	1.9 (0.0, 4.7)	8	0.6 (0.0, 1.2)	1.3 (− 0.7, 4.1)
Income (low-income cut-off)							
Above	188	13.1 (9.3, 16.9)	30	8.5 (3.2, 13.7)	154	14.3 (9.8, 18.8)	− 5.8 (− 12.8, 1.1)
Below	713	86.9 (83.1, 90.7)	121	91.5 (86.3, 96.8)	570	85.7 (81.2, 90.2)	5.8 (− 1.1, 12.8)
Employment status							
Employed	273	18.2 (13.6, 22.8)	31	8.4 (0.0, 16.8)	235	22.0 (16.5, 27.5)	− 13.6 (− 23.6, − 3.6)*
Unemployed	473	63.0 (57.4, 68.6)	112	81.4 (71.2, 91.5)	347	55.9 (49.3, 62.4)	25.5 (13.4, − 37.5)*
Not in labour force	135	18.8 (14.3, 23.3)	6	10.2 (3.8, 16.6)	125	22.1 (16.7, 27.6)	− 11.9 (− 20.3, − 3.5)*
Education level							
Some high school or less	390	49.4 (43.7, 55.1)	83	52.0 (38.1, 66.0)	294	48.5 (42.2, 54.8)	3.5 (− 11.8, 18.9)
Completed high school	171	17.9 (13.2, 22.7)	33	14.0 (2.4, 25.6)	130	19.1 (13.9, 24.3)	− 5.1 (− 17.8, 7.6)
Some or completed college	226	24.8 (20.1, 29.5)	27	29.7 (18.3, 41.0)	196	23.7 (18.3, 29.0)	6.0 (− 6.6, 18.5)
Some or completed university	126	7.8 (4.8, 10.8)	11	4.3 (1.0, 7.6)	112	8.7 (5.1, 12.4)	− 4.4 (− 9.4, 0.6)

^*^Significant difference in proportions at the 95% confidence level

^a^Non-responses (*n* = 27) to the household question used to measure physical homelessness were treated as missing values and were not included in the stratified analysis

**Table 3:** Describing the 12 dimensions of Indigenous homelessness (Thistle, 2017) experienced by FNIM adults in Toronto

Dimensions of homelessness (Thistle, 2017)	FNIM adults in Toronto (*n* = 915)		FNIM adults living physically homeless (*n* = 154)^b^		FNIM adults living physically housed (*n* = 734)^b^		Difference in proportions using RDS MOVER % (95% CI)
	*n*	RDS-II % (95% confidence interval)	*n*	RDS-II % (95% confidence interval)	*n*	RDS-II % (95% confidence interval)	
Historic displacement	[*Not measurable using current data source*]						
Contemporary geographic separation	596	58.5 (52.9, 64.1)	98	76.3 (64.3, 88.3)	481	51.6 (45.4, 57.9)	20.3 (8.1, 32.2)*
Spiritual disconnection	378	49.8 (44.1, 55.5)	67	40.9 (27.0, 54.7)	298	52.3 (46.0, 58.7)	− 11.4 (− 26.7, 3.8)
Mental disruption and imbalance	584	71.5 (66.3, 76.78)	115	86.4 (76.3, 96.5)	449	65.6 (59.6, 71.6)	20.8 (9 .1, 32.5)*
Cultural disintegration and loss	803	84.4 (80.8, 88.0)	140	94.0 (88.3, 99.7)	641	80.6 (76.3, 84.8)	13.4 (6.3, 20.5)*
Overcrowding^a^	71	13.8 (10.3, 17.3)	-	-	52	8.3 (4.8, 11.9)	-
Relocation and mobility	352	52.1 (46.3, 57.8)	108	78.5 (67.7, 89.2)	230	42.3 (36.1, 48.6)	36.2 (23.7, 48.6)*
Going home	[*Not measurable using current data source*]						
Nowhere to go^b^	154	27.3 (22.7, 31.9)	-	-	-	-	-
Escaping or evading harm	[*Not measurable using current data source*]						
Emergency crisis	[*Not measurable using current data source*]						
Climate refugee	[*Not measurable using current data source*]						

^*^Significant difference in proportions at the 95% confidence level

^a^Details required to calculate overcrowding were only available for a subset of the population who reported living physically housed

^b^The same measure is used for nowhere to go homelessness and physical homelessness. Non-responses (*n* = 27) to the household question used to measure nowhere to go homelessness/physical homelessness were treated as missing values and were not included in the stratified analysis

#### Historic displacement

“Historic displacement” refers to how Indigenous communities were historically removed and displaced from their lands through colonization. Through implementation of colonial projects such as The Reserve System, The Pass System, Métis Scrip, and the Inuit High Arctic Relocations, Indigenous people have been alienated from their lands and resources and consequently disconnected from their traditional territories, cultural practices, and traditions (Thistle, 2017). OHC does not have questions that measure historic displacement.

#### Contemporary geographic separation

Similar to historic displacement homelessness, “contemporary geographic separation” refers to the historic colonial projects that removed Indigenous communities from their pre-colonial lands. However, this dimension also considers the impact of Canadian policies against Indigenous people, such as the Indian act, residential schools, and the Sixties Scoop and how this ongoing colonial legacy has geographically displaced Indigenous populations from their connection to traditional lands, resources, and communities (Thistle, 2017). OHC questions on residential school attendance, experiences with the Sixties Scoop, and experiences of racism/discrimination were used (Table 1). All questions had binary responses. Participants who responded “yes” to one or more of the above questions were categorized as experiencing contemporary geographic separation.

#### Spiritual disconnection

“Spiritual disconnection” refers to Indigenous worldviews and understandings of home as being a way of life more than being a physical place (Christensen, 2013). This dimension assesses how, through colonization, Indigenous people have been separated from their spiritual belief systems, including worldviews, traditions, ceremonies, stories, and teachings, thereby hindering the practice and knowledge of Indigenous culture. This separation can damage the Indigenous spirit, which contributes to a disconnection from identity, community, family, and participation in cultural activities. Measures pertaining to participating in Indigenous ceremonies, use of traditional medicines, ability to speak Indigenous languages, and consumption of traditional foods were used (Table 1). All questions had binary responses or were recoded to have a binary categorization. Participants who responded “no” to one or more of the above were categorized as experiencing spiritual disconnection.

#### Mental disruption and imbalance

“Mental disruption and imbalance” refers to “the loss of healthy mental functioning” (Thistle, 2017, pg. 34) due to Canadian policies against Indigenous people and the impact of discrimination, racism, marginalization, and exclusions from Canadian settler society. As a result, high rates of mental health and psychological challenges, substance use, and family violence exist in Indigenous communities. These issues are often passed on through generations, and experiences of intergenerational trauma put people at elevated risk of homelessness (Thistle, 2017). OHC questions about mental health diagnoses, the Kessler Psychological Distress Scale (K-10), and the Primary Care PTSD Screen (PC-PTSD) were used to measure this dimension. Detailed descriptions of how the K-10 and PC-PTSD measures were used in OHC are published elsewhere (Firestone et al., 2022). Participants who reported having a mental health diagnosis or who screened positive to depression or PTSD were categorized as experiencing mental disruption and imbalance.

#### Cultural disintegration and loss

“Cultural disintegration and loss” refers to the alienation and dislocation experienced by Indigenous individuals due to loss of connection to culture, identity, family and kinship networks, and the broader community. Similar to spiritual disconnection homelessness, this dimension considers the loss of knowledge and practice of Indigenous culture that was largely influenced by discriminatory colonial policies against Indigenous people; however, this dimension further considers how this ongoing colonial legacy has led to cultural shame and limited access to cultural traditions, gatherings, ceremonies, songs, activities, family and kinship networks, and Indigenous languages (Thistle, 2017). Questions about barriers to accessing ceremonies, access to traditional foods, connection to Mother Earth, and kin and social relationships were used (Table 1). Participants who indicated they had limited access to one or more of the above were categorized as experiencing cultural disintegration and loss.

#### Overcrowding

“Overcrowding” refers to the number of people living in a household that exceeds the national average (Statistics Canada, 2019). Living in overcrowded households has been linked to experiences of unsafe living spaces, poor health standards, family strain, eviction, financial stressors, crisis situations, and substance use, thereby increasing the risk of physical homelessness (Thistle, 2017). Questions about number of people and number of rooms in the household were used to calculate a derived variable of overcrowding. Using Statistics Canada’s measures of overcrowding (Statistics Canada, 2019), overcrowding was defined as more than one person per room living in a household.

#### Relocation and mobility

“Relocation and mobility” refers to the increased experiences of homelessness or housing instability among Indigenous people who frequently travel or move across geographies (Thistle, 2017). Indigenous people are highly mobile (Belanger, 2013; Firestone et al., 2018) and may become mobile or relocate for various reasons, including access to work, education, kin and social networks, health care, housing (Firestone et al., 2018; Snyder & Wilson, 2012), childcare, recreation, or cultural events (Thistle, 2017). Questions pertaining to frequency of movement in the past year were used to measure relocation and mobility. Participants who indicated they had moved one or more times in the past year were categorized as experiencing relocation and mobility.

#### Going home

“Going home” homelessness refers to how Indigenous people may experience a decreased sense of “home” and belonging to their community due to being raised outside of their community or being away for a prolonged period of time for reasons such as education or employment opportunities. These experiences create barriers to accessing housing in their home community and thereby hinder the ability to return home (Thistle, 2017). The OHC survey tool does not have questions related to going home homelessness.

#### Escaping or evading harm

“Escaping or evading harm” refers to how Indigenous populations experience housing instability or homelessness due to the need to escape unsafe living environments or harmful situations, such as domestic violence or experiences of racism and discrimination. This dimension considers intersecting factors such as sexual orientation, gender, and age as 2SLGBTQ + persons, youth, and women experience increased instances of violence (Thistle, 2017). There are no questions on the OHC survey tool that measure escaping or evading harm homelessness.

### Emergency crisis

The dimension of “emergency crisis” refers to experiences of housing instability or homelessness due to natural disasters, a breakdown of social supports, and systemic issues such as racism, government mandates, and an absence of emergency plans from formal institutions that support Indigenous populations during crises (Thistle, 2017). OHC does not have questions related to experiences of emergency crisis homelessness.

#### Climate refugee

“Climatic refugee” homelessness refers to the impact of displacement and housing instability due to changing climatic conditions. This dimension measures how Indigenous populations are specifically impacted by climate change, such as increased food scarcity on traditional lands (Thistle, 2017). OHC does not have questions that measure climate refugee forms of homelessness.

### Statistical analysis

All data cleaning and coding was completed in SAS 9.4 statistical software (SAS Institute Inc., 2013). All descriptive analyses were completed in R Software using the RDS package (R Core Team, 2013). RDS-II adjusted proportions and 95% confidence intervals were produced to describe specific sociodemographic characteristics of the entire population and to calculate proportions and counts of FNIM adults who had experienced the *measurable* dimensions of Indigenous homelessness. The distribution of specific sociodemographic characteristics and the dimensions of homelessness were compared between FNIM adults who were and those who were not experiencing physical homelessness. Difference in proportions and 95% confidence intervals were calculated using the method of variance estimates recovery (MOVER) method (Rotondi, 2014).

## Results

In terms of sociodemographic characteristics, more than one quarter of FNIM adults in Toronto experienced “nowhere to go homelessness/physical homelessness” (27.3%; 95% CI, 22.7, 31.9). Among FNIM adults living physically homeless, most identified as male (65.7%; 95% CI, 52.6, 78.7) and were between the ages of 26 and 54 (79.7%; 95% CI, 69.3, 90.2). The proportion of males was significantly higher among the population living physically homeless compared to the population not living physically homeless. Most FNIM adults living physically homeless were also living below the low-income cut-off (LICO) (91.5%; 95% CI, 86.3, 96.8), were unemployed (81.4%; 95% CI, 71.2, 91.5), and had educational attainment of some high school or less (52.0%; 95% CI, 38.1, 66.0). Some FNIM adults living homeless were living above the LICO (8.5%; 95% CI, 3.2, 13.7) and had some college training (29.7%; 95% CI, 18.3, 41.0); however, LICO and educational attainment levels were similar among the population living physically homeless and the population not living physically homeless (Table 2).

Seven of the twelve dimensions of Indigenous homelessness were measurable using the OHC dataset. Among all FNIM adults in Toronto (i.e., those reporting and those not reporting physical homelessness), most experienced “cultural disintegration and loss” (84.4%; 95% CI, 80.8, 88.0), “mental disruption and imbalance” (71.5%; 95% CI, 66.3, 76.78), “contemporary geographic separation” (58.5%; 95% CI, 52.9, 64.1), and “relocation or mobility” (52.1%; 95% CI, 46.3, 57.8) forms of homelessness. Almost half of FNIM adults in Toronto experienced “spiritual disconnection” homelessness (49.8%; 95% CI, 44.1, 55.5) (Table 3).

Experiences of “cultural disintegration and loss” (94.0%; 95% CI, 88.3, 99.7), “mental disruption and imbalance” (86.4%; 95% CI, 76.3, 96.5), “contemporary geographic separation” (76.3%; 95% CI, 64.3, 88.3), and “relocation and mobility” (78.5%; 95% CI, 67.7, 89.2) forms of homelessness were significantly higher among FNIM adults living physically homeless in Toronto in comparison with those who were not living physically homeless. Among FNIM living housed, 13.8% (95% CI, 10.3, 17.3) were living in overcrowded households (Table 3).

Almost all FNIM adults in Toronto experienced one or more of the seven measurable dimensions of homelessness. After removing missing data, only 0.2% (95% CI, 0.1, 0.4) of FNIM adults in Toronto did not experience any of the dimensions of homelessness, whereas 38.9% (95% CI, 32.2, 45.5) experienced three dimensions, 26.2% (95% CI, 20.5, 32.0) experienced two, and 21.9% (95% CI, 15.9, 27.8) experienced four. No one experienced all seven (Table 4).

**Table 4:** The number of the 12 dimensions of Indigenous homelessness (Thistle, 2017) experienced by FNIM adults in Toronto

Number of dimensions of Indigenous homelessness (Thistle, 2017) experienced	FNIM adults in Toronto (*n* = 641)^a^	
	*n*	RDS-II % (95% confidence interval)
0	-	0.2 (0.1, 0.4)
1	51	5.5 (2.6, 8.5)
2	158	26.2 (20.5, 32.0)
3	237	38.9 (32.2, 45.5)
4	151	21.9 (15.9, 27.8)
5	36	6.9 (3.9, 9.9)
6	-	-
7	0	-

^a^Population after excluding missing data on any of the 7 measurable dimensions of homelessness

## Discussion

This study builds on current research that shows high rates of physical homelessness among Indigenous people living in urban and related homelands (Belanger, 2013; Brar et al., 2020; Firestone et al., 2018). Study findings also show that specific sociodemographic factors contribute to the Indigenous experience of homelessness. While education levels were similar among those FNIM adults who were living physically homeless and those not living physically homeless, there were significantly higher proportions of FNIM adults who were unemployed or not in the labour force among FNIM adults living physically homeless as compared with those not living homeless.

Importantly, the findings from this study indicate that there were high rates of 7 of the 12 dimensions of Indigenous homelessness experienced by FNIM adults in Toronto. By applying Thistle’s (2017) Indigenous homelessness framework to an Indigenous population database, we determined that the most common dimensions experienced by FNIM adults in Toronto (those experiencing and those not experiencing physical homelessness) were those of cultural disintegration and loss, mental disruption and imbalance, and contemporary geographic separation. These dimensions were significantly higher among FNIM adults living physically homeless. The relocation and mobility form of homelessness was also common; more than half of FNIM adults reported moving one or more times in the past year. This finding is consistent with previous research that shows high mobility among Indigenous populations (Belanger, 2013; Firestone et al., 2018; Snyder & Wilson, 2012). Experiences of mobility and relocation are closely tied to experiences of physical homelessness, as mobility and relocation were significantly higher among FNIM adults living physically homeless. Proportions of contemporary geographic separation, cultural disintegration and loss, mental disruption and imbalance, and relocation and mobility forms of homelessness were all significantly higher among FNIM adults experiencing homelessness, thereby providing quantitative evidence that Indigenous populations have unique experiences that influence pathways into physical homelessness.

Western understandings of homelessness define homelessness as being without a physical habitation structure. This is only one of several forms of homelessness that Indigenous people may experience. According to Thistle (2017), there are 11 additional forms of homelessness that need to be considered. Thistle (2017) hypothesizes that all Indigenous people have experienced one or more of the 12 dimensions of homelessness in their lifetime. In the case of this study, 7 of the 12 dimensions of Indigenous homelessness were measurable using the OHC dataset. The findings from this study indicate that almost all participants had experienced one or more of the seven measurable dimensions of homelessness. While this evidence is important in developing a deeper understanding of these dimensions, it also points to a clear gap in our understanding of how the remaining dimensions of homelessness impact Indigenous people living in urban and related homelands.

To fully understand the Indigenous experience of homelessness and unique factors that impact pathways into homelessness among this population, a survey tool that includes measures for each of the 12 dimensions of Indigenous homelessness is required. Currently, a survey tool of this nature does not exist. Conventional measures used to describe homelessness for Indigenous and non-Indigenous populations ignore the impacts of colonialism and the unique understandings and experiences that pertain to homelessness among Indigenous individuals, families, and communities. The need for a homelessness indicator rooted in Indigenous epistemologies has created a significant gap in knowledge regarding how Indigenous homelessness is defined, counted, and understood. Given the high rates of homelessness among Indigenous peoples in urban and related homelands, compounded by the impacts of systemic racism, there remains a pressing need to improve qualitative and quantitative measures of Indigenous homelessness, increase the reliability of population data, provide culturally safe services to Indigenous populations, and address the underlying causes of homelessness (McCallum & Isaac, 2011). The creation of a new survey tool that measures homelessness through an Indigenous lens is imperative to providing a culturally relevant evidence base of the scope and severity of the Indigenous experience of homelessness, and will allow us to produce data that estimate the historical, environmental, social, political, spiritual, and emotional factors that lead into pathways of homelessness.

The high rates of physical homelessness and other dimensions of homelessness observed in this study can be used to understand the unique needs of Indigenous people experiencing homelessness and inform the gaps in health and social services dedicated to supporting Indigenous peoples living in Toronto. This research provides evidence that the residential school system, experiences of racism and discrimination, mental health challenges, cultural disenfranchisement and limited access to culture, and mobility are unique factors among Indigenous people that influence pathways into homelessness. This points to a critical need to develop more Indigenous-specific housing supports in Toronto, with programs dedicated to access to cultural activities, language, food, mental wellness and addictions recovery, financial literacy and management, and education and employment development. Wraparound services that meet the holistic needs of Indigenous people and promote connection to land, culture, language, and community are required to interrupt pathways into homelessness among Indigenous people.

### Study limitations

This study has some limitations. The data source used for this study was not initially developed with the intention of measuring the 12 dimensions of Indigenous homelessness. As such, some dimensions could not be assessed in this study. In addition, some questions may not fully capture or reflect the extent of each dimension. For example, there are aspects of contemporary geographic separation, spiritual disconnection, cultural disintegration and loss, and mental disruption and imbalance that could not be assessed using the available indicators in the data source. These limitations could be addressed by future research that focuses on developing and validating a new survey tool that measures Indigenous homelessness using Indigenous worldviews and conceptualizations of homelessness.

## Conclusion

This study applied Thistle’s (2017) 12 dimensions of Indigenous homelessness framework to an Indigenous population database to generate population-based estimates of the proportion of FNIM adults experiencing physical homelessness and six other forms of homelessness. FNIM adults in Toronto experienced high rates of physical homelessness and experiences of contemporary geographic separation, spiritual disconnection, cultural disintegration and loss, mental disruption and imbalance, and relocation and mobility forms of homelessness. Five of the twelve dimensions could not be assessed using the available data source. To accurately measure and understand the unique experiences of homelessness impacting Indigenous people living in urban and related homelands, a new population-level survey tool with measures that fully represent and capture the extent of all 12 of the dimensions of Indigenous homelessness is required. These findings also point to the need for responsive policies that will address the disproportionate experiences of homelessness among Indigenous peoples in Toronto and beyond. This includes adequate funding allocations and culturally relevant and holistic services that support Indigenous people experiencing homelessness across the various dimensions, including addressing associated factors such as gaps in access to employment and education.

## Contributions to knowledge

What does this study add to existing knowledge?

- Thistle’s (2017) 12 dimensions of Indigenous homelessness framework was applied to an Indigenous-owned population health database to generate population estimates for the prevalence of physical homelessness and six other dimensions of Indigenous homelessness among FNIM adults in Toronto.
- Our Health Counts is a one of its kind, Indigenous-owned population health database. For this project, the OHC Toronto database was used to uncover alarming gaps in how homelessness is quantified and measured. Only 7 of the 12 dimensions of Indigenous homelessness were measurable using the available data source, pointing to a critical need to develop an Indigenous-led survey tool that appropriately measures population levels of homelessness among Indigenous peoples living in urban and related homelands.

What are the key implications for public health interventions, practice, or policy?

- There are unique pathways into homelessness among the FNIM population living in urban and related homelands, including social, cultural, environmental, historical, and political factors.
- A new survey tool that measures all dimensions of Indigenous homelessness is needed to appropriately address the unique needs of FNIM peoples experiencing homelessness.

## Data Availability

The OHC Toronto data are owned and governed by community partners, Seventh Generation Midwives Toronto. A data sharing and publication agreement exists that details the data governance protocols and the roles and responsibilities of staff, students, and other researchers who request to use the OHC Toronto data.

## Code availability

N/A.
